# Artifact of “Breakthrough” osmosis: comment on the local Spiegler-Kedem-Katchalsky equations with constant coefficients

**DOI:** 10.1038/s41598-021-83404-9

**Published:** 2021-03-03

**Authors:** Isaak Rubinstein, Amnon Schur, Boris Zaltzman

**Affiliations:** Blaustein Institutes for Desert Research, Ben-Gurion University of the Negev, Be’er Sheva, Israel

**Keywords:** Devices for energy harvesting, Chemical engineering

**arising from**: A. Yaroshchuk; *Scientific Reports* 10.1038/srep45168 (2017).

## Introduction

**Figure 1:**
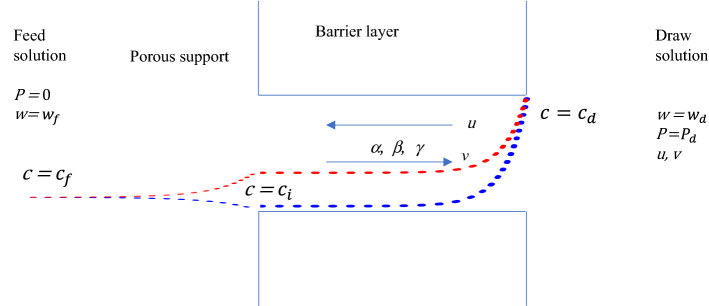
Solute concentration profiles in the breakthrough mode following from SKK with constant coefficients (blue) and MSKK (red): *c*—solute concentration, *c*_f_—feed concentration, *c*_d_ —draw concentration, *P*_d_—draw pressure; Capillary friction model: *u*—solute velocity, *v*—volume (solvent) velocity, *α*,*β*,*γ*—friction coefficients.

Osmosis—solution (solvent) flow through non-perfectly (perfectly) semipermeable membranes—is a fundamental classical phenomenon of major practical importance. One of its potentially useful technological applications is the Pressure Retarded Osmosis (PRO) employed for energy harvesting from salinity variations^1^. In this process the flow resulting from the osmotic pressure drop between fresh and saline water is used to drive a turbine. Unfortunately, at the current stage, in spite of its extreme simplicity and conceptual beauty, this process does not appear to be practically viable due to insufficient power efficiency^2^. This assessment could be radically changed by the recently theoretically predicted “Breakthrough” operation mode of PRO^3^. In this mode, the solute concentration at the interface between the porous support and the dense selective barrier layer of a non-perfect (‘leaky’) asymmetric membrane employed in PRO decreases with the increase of draw concentration, and, thus, the impeding effect of internal concentration polarization is eliminated, Fig. 1. The existence of this mode was predicted by Yaroshchuk based on the accurate analysis of the system of classical local Spiegler-Kedem-Katchalsky (SKK) equations of membrane transport with three constant coefficients for the barrier layer: solute permeability (diffusivity), solute reflection coefficient and hydraulic permeability^4^. In a still more recent study Wu and Field^5^, contested the physical feasibility of “Breakthrough mode” and casted doubt upon the suitability of SKK equations with constant coefficients to PRO. In this note we re-derive the SKK equations based on a very simple capillary friction model of membrane transport in the dense barrier layer and identify the problem with the constant coefficients’ assumption resulting in the occurrence of “Breakthrough mode”. Our derivation results in recovering the SKK equations in the dilute solution limit, albeit with hydraulic permeability dependent on the local solute concentration in the barrier layer (modified SKK equations, MSKK). Taking into account this dependence, necessary for preserving the detailed force balance in the barrier layer, eliminates the existence of the “Breakthrough mode”.

## Results and conclusions

**Figure 2:**
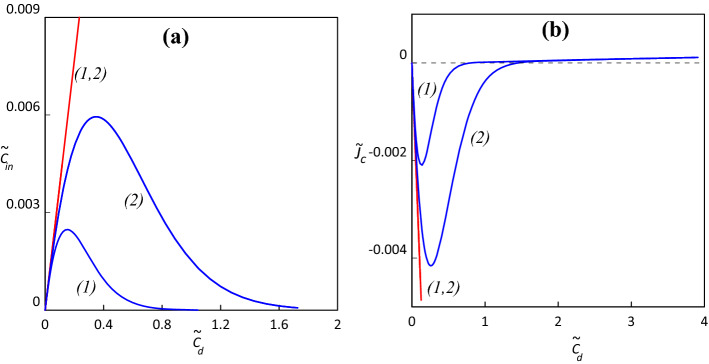
The dependence of the interface concentration ${\tilde{c}}_{in}$ (**a**) and solute flux ${\tilde{J}}_{c}$ (**b**) on the draw concentration ${\tilde{c}}_{d}$ for classical SKK (blue line) and modified MSKK (red line) equations, (1) $\sigma =0.8$, (2) $\sigma =0.9$.

In Fig. 2a,b we present the dependence of the interface concentration and solute flux on the draw concentration in classical SKK and MSKK model.

We observe that in MSKK setup the “Breakthrough” mode, typified by the non-monotonic dependence of the interface solute concentration on the draw concentration and the accompanying possible reversal of the sign of the solute flux, disappears. As shown in the methods section, interface concentration is a monotonically increasing function of draw concentration independently of the specific parameters’ values chosen for estimates, Table 1. This follows from the dependence of the hydraulic permeability in the MSKK model on the local solute concentration in the barrier layer in accord with the capillary friction model. This dependence is necessary for preserving the detailed local overall force balance in the barrier layer. Violation of this balance in the classical local SKK model with constant coefficients results in the artifact of “Breakthrough” mode. This mode has been predicted in^3^ based on accurate analysis of the SKK model with constant coefficients for a particular narrow range of operation parameters (a very low feed concentration $, {\tilde{c}}_{f} <1-\sigma$*,* and a very high draw concentration, ${\tilde{c}}_{d}$). Finally, we note that the difference between MSKK and SKK manifests itself only for exceedingly high concentrations for which the very usage of the high dilution limit is questionable. For low/moderate concentrations the local SKK model with constant coefficients appears to be a valid approximation.

**Table 1:** Values of parameters employed in computations.

*D*	${10}^{-5}{\mathrm{cm}}^{2}/\mathrm{s}$
ω	${10}^{-8}\frac{{\mathrm{cm}}^{2}}{\mathrm{s}}$
$\sigma$	0.9 (1), 0.8 (2)
$\gamma =\frac{RT}{\omega }$	$2.5\cdot {10}^{15}\frac{\mathrm{kg}}{\mathrm{mol}\cdot \mathrm{s}}$
*w*	$0.05\frac{\mathrm{mol}}{{\mathrm{cm}}^{3}}$
*c*	${10}^{-5}\frac{\mathrm{mol}}{{\mathrm{cm}}^{3}}$
$\delta$	$5\cdot {10}^{-4}\mathrm{cm}$
$l$	$2\cdot {10}^{-2}\mathrm{cm}$
$\frac{\chi }{\delta }$	$9.2\cdot {10}^{-8}\frac{{\mathrm{cm}}^{2}\cdot \mathrm{s}}{\mathrm{kg}}$
$\chi$	$4.6\cdot {10}^{-13}\frac{{\mathrm{cm}}^{3}\cdot \mathrm{s}}{\mathrm{kg}}$
$\alpha$	$2.2\cdot {10}^{14}\frac{\mathrm{kg}}{\mathrm{mol}\cdot \mathrm{s}}$

## Methods

### Capillary friction model

Following the common pattern of friction models^6,7^, we neglect inertia and assume linear friction of solute with water and each of them with the immobile capillary wall. Here, the term capillary is just a figure of speech standing for the solid matrix of the dense barrier layer. With these assumptions, assuming in addition ideal solution and using the ideal gas mixture equation as the simplest model for it, the local force balance in the capillary for each solution component per unit volume reads, Fig. 1:

1 $${\text{Solute}}:\quad wc\beta \left( {u - v} \right) + c\gamma u = - c\Omega_{c} \frac{{{\text{d}}P}}{{{\text{d}}x}} - cRT\frac{{{\text{d}}\left( {{\text{ln}}\frac{c}{c + w}} \right)}}{{{\text{d}}x}}$$

2 $${\text{Water}}:\quad wc\beta \left( {v - u} \right) + w\alpha v = - w\Omega_{w} \frac{{{\text{d}}P}}{{{\text{d}}x}} - wRT\frac{{{\text{d}}\left( {{\text{ln}}\frac{w}{w + c}} \right)}}{{{\text{d}}x}}$$

here, $x$ is a longitudinal coordinate along the capillary, *u* and *v* are the velocities of solute and water (this latter is equivalent for a dilute solution assumed heron to the volume flux of solution), $\alpha , \gamma , \beta$—friction coefficients of a water molecule with the wall, solute molecule with the wall, and mutual friction between both molecules; *c*(*x*), and *w*(*x*) are the solute and water number densities and *P*(*x*) is hydrostatic pressure; ${\Omega }_{w}$ and ${\Omega }_{c}$ are constant molecular volumes of water and solute. The following equality holds:

3 $${\Omega }_{c}c+{\Omega }_{w}w=1$$

Summation of (1) and (2), taking into account (3), yields the overall detailed momentum balance in the form:

4 $$c\gamma u+\alpha wv=-\frac{\mathrm{d}P}{\mathrm{d}x}$$

Here, the identity

5 $$c\frac{\mathrm{d}\left(\mathrm{ln}\frac{c}{c+w}\right)}{\mathrm{d}x}+w\frac{\mathrm{d}\left(\mathrm{ln}\frac{w}{w+c}\right)}{\mathrm{d}x}=0$$

has been used. This identity is a particular trivial version of the Gibbs–Duhem equation.

Equation (4) implies that the pressure gradient in the capillary is balanced by the friction of the solution components with the capillary walls.

Defining the spatially constant water and solute molecular fluxes as ${J}_{w}=wv$, and ${J}_{c}=cu$*,* and referring to high dilution $(c\ll w, w=1/{\Omega }_{w})$ Eqs. (1), (4) assume their final form:

6 $$\beta {wJ}_{c}-\beta c{J}_{w}+\gamma {J}_{c}={-c\Omega }_{c}\frac{\mathrm{d}P}{\mathrm{d}x}-cRT\frac{\mathrm{d}\left(\mathrm{ln}\frac{c}{c+w}\right)}{\mathrm{d}x}\cong {-c\Omega }_{c}\frac{\mathrm{d}P}{\mathrm{d}x}-RT\frac{\mathrm{d}c}{\mathrm{d}x}$$

7 $$\gamma {J}_{c}+\alpha {J}_{w}=-\frac{\mathrm{d}P}{\mathrm{d}x}$$

Equations (6), (7) may be rewritten as:

8 $${J}_{c}=-\omega {c}_{x}+(1-\sigma [1-\frac{\alpha }{\gamma }\frac{{\Omega }_{c}}{{\Omega }_{w}}])cv$$

9 $$v=-\chi ({P}_{x}-\sigma RT{c}_{x})$$

here,

10 $$\omega =\frac{RT}{\beta w+\gamma }$$

is solute diffusivity,

11 $$\sigma =\frac{\gamma }{\beta w+\gamma }$$

is the Staverman’s solute reflection coefficient, and

12 $$\chi =\frac{1/w}{\alpha +\gamma \frac{(\beta +\alpha {\Omega }_{c})c}{\beta w+\gamma }}$$

is hydraulic permeability of the barrier layer.

For ${\alpha \Omega }_{c}\ll \gamma {\Omega }_{w}$ Eqs. (8) is reduced to

13 $${J}_{c}=-\omega {c}_{x}+(1-\sigma )cv.$$

Equations (9), (13) are the SKK equations (adhering to the notations in^3^), albeit with hydraulic permeability dependent on the solute concentration in the capillary (barrier layer) in accord with (12). So modified SKK equations preserve the overall detailed momentum balance in the capillary (4), as opposed to SKK model with constant coefficients. These modified SKK equations (MSKK) are employed in the following subsection to prove the non-existence of the breakthrough mode.

### Integration of MSKK equations and absence of breakthrough mode

In the porous support the dimensionless solute flux reads

14 $$\tilde{J}_{c} = - \frac{{{\text{d}}\tilde{c}}}{{{\text{d}}\tilde{x}}} + \tilde{c}\tilde{v},\;\;\; - 1 < \tilde{x} < 0$$

where $\tilde{x}=\frac{x}{l},$ $l$ is the support layer thickness; $\tilde{c}=\frac{c}{w}$ , $\tilde{v}=\frac{v}{{v}_{0}}, {v}_{0}=\frac{D}{l}$ , $D$ is solute diffusivity in the porous support and ${\tilde{J}}_{c}=\frac{{J}_{c}l}{Dw}$.

Integrating Eq. (14), we compute the solute flux and the dimensionless solute concentration in the porous support in the form:

15 $${\tilde{J}}_{c}=({\tilde{c}}_{f}{e}^{\tilde{v}}-{\tilde{c}}_{in})\frac{\tilde{v}}{{e}^{\tilde{v}}-1}$$

here, ${\tilde{c}}_{f}=\tilde{c}(-1)$ and ${\tilde{c}}_{in}=\tilde{c}(0)$ for the solute concentration at the interface between the porous support and barrier layer.

Integration of the dimensionless flux equation in the latter

16 $$\tilde{J}_{c} = - \tilde{\omega }\frac{{{\text{d}}\tilde{c}}}{{{\text{d}}\tilde{x}}} + \left( {1 - \sigma } \right)\tilde{c}\tilde{v},\;\;\;0 < \tilde{x} < {\Delta },$$

where $\Delta =\frac{\delta }{l}$ is the dimensionless counterpart of the barrier layer’s thickness $\delta$ and $\tilde{\omega }=\frac{\omega }{D}$, yields

17 $${\tilde{c}}_{d}=\tilde{c}\left(\Delta \right)=\frac{{\tilde{J}}_{c}}{(1-\sigma )\tilde{v}}+\left({\tilde{c}}_{in}-\frac{{\tilde{J}}_{c}}{(1-\sigma )\tilde{v}}\right){e}^{\frac{1-\sigma }{\tilde{\omega }}\tilde{v}\Delta }$$

To complete the formulation, we prescribe the pressure drop $\Delta P$ across the barrier layer, and for the case of non-retarded osmosis, $\Delta P=0,$ obtain referring to the detailed force balance Eqs. (4), (7):

18 $$\tilde{J}_{c} = - \tilde{\alpha }\tilde{v},$$

where $\tilde{\alpha }=\frac{\alpha }{\gamma }$. The independence of the $\tilde{J}_{c}\ \mathrm{to}\ \tilde{v}$ ratio of concentration implied by (18) stands in accord with the available experimental data on Forward Osmosis (see^8^, Fig. 5 and^9^, Fig. 5 therein). Substituting (18) into Eqs. (15), (17) we find

19 $$\tilde{c}_{in} - \tilde{c}_{f} e^{{\tilde{v}}} = \tilde{\alpha }(e^{{\tilde{v}}} - 1),$$

20 $${\tilde{c}}_{d}=-\frac{\tilde{\alpha }}{1-\sigma }+\left({\tilde{c}}_{in}+\frac{\tilde{\alpha }}{1-\sigma }\right){e}^{\frac{1-\sigma }{\tilde{\omega }}\tilde{v}\Delta }$$

and, thus, the dimensionless velocity is subject to the following algebraic equation

21 $$\tilde{c}_{d} = - \frac{{\tilde{\alpha }}}{1 - \sigma } + \left( {\tilde{c}_{f} e^{{\tilde{v}}} + \tilde{\alpha }e^{{\tilde{v}}} + \frac{{\tilde{\alpha }\sigma }}{1 - \sigma }} \right)e^{{\frac{1 - \sigma }{{\tilde{\omega }}}\tilde{v}{\Delta }}} = F\left( {\tilde{v}} \right).$$

Since $\frac{\mathrm{d}F}{\mathrm{d}\stackrel{\sim }{v}}>0$, the flow velocity $\tilde{v}$ is a monotonically increasing function of the draw concentration ${\tilde{c}}_{d}$ and, therefore, the interface concentration ${\tilde{c}}_{in}={\tilde{c}}_{f}{e}^{\tilde{v}}+\tilde{\alpha }{(e}^{\tilde{v}}-1)$ is an increasing function of ${\tilde{c}}_{d}$ too. This proves the non-existence of the breakthrough mode in the MSKK model as a result of preserving the detailed force balance in the barrier layer.

For high concentrations, ${\tilde{c}}_{d}\gg 1,$ the solution of the Eqs. (19), (21) reads

22 $$\tilde{v}=\frac{\omega }{\omega +(1-\sigma )\Delta }\mathrm{ln}{\tilde{c}}_{d}$$

23 $${\tilde{c}}_{in}=(\tilde{\alpha }+{\tilde{c}}_{f}){{\tilde{c}}_{d}}^{\frac{\omega }{\omega +(1-\sigma )\Delta }}$$

In Fig. 2a, b we illustrate the dependence of the interface concentration and solute flux on the draw concentration in both classical SKK and modified MSKK models.

The Modified Spiegler-Kedem-Katchalsky (MSKK) model is derived based on the capillary friction model. The detailed force balance in this model yields a solute concentration dependent hydraulic permeability and eliminates the breakthrough mode predicted by the classical local SKK equations with constant coefficients. The difference between the MSKK and the SKK models manifests itself only for extremely high solute concentration range for which the occurrence of breakthrough mode has been predicted.

## References

[1] Loeb S, Norman RS (1975). Osmotic power plants. Science.

[2] Straub AP, Deshmukh A, Elimelech M (2016). Pressure-retarded osmosis for power generation from salinity gradients: is it viable?. Energy Environ. Sci..

[3] Yaroshchuk A (2017). “Breakthrough” osmosis and unusually high power densities in pressure-retarded osmosis in non-ideally semi-permeable supported membranes. Sci. Rep..

[4] Spiegler KS, Kedem O (1966). Thermodynamics of hyperfiltration (reverse osmosis): criteria for efficient membranes. Desalination.

[5] Wu JJ, Field RW (2019). On the understanding and feasibility of “Breakthrough” osmosis. RSci. Rep..

[6] Spiegler KS (1958). Transport processes in ionic membranes. Trans. Faraday Soc..

[7] Kerkhof PJAM (1996). A modified Maxwell–Stefan model for transport through inert membranes: the binary friction model. Chem. Engl. J..

[8] Phillip WA, Yong JS, Elimelech M (2010). Reverse draw solute permeation in forward osmosis: modeling and experiments. Environ. Sci. Technol..

[9] Yong JS, Phillip WA, Elimelech M (2012). Coupled reverse draw solute permeation and water flux in forward osmosis with neutral draw solutes. J. Membr. Sci..

